# Supplementary material for: GOcats: A tool for categorizing Gene Ontology into subgraphs of user-defined concepts
Source: PLoS One. 2020 Jun 11;15(6):e0233311. doi: 10.1371/journal.pone.0233311 (PMC7289357; doi:10.1371/journal.pone.0233311)

**Supplementary Data 1 – Visualizing the degree of overlap between the category subgraphs created by GOcats, Map2Slim, and the UniProt CV (additional categories)**

**
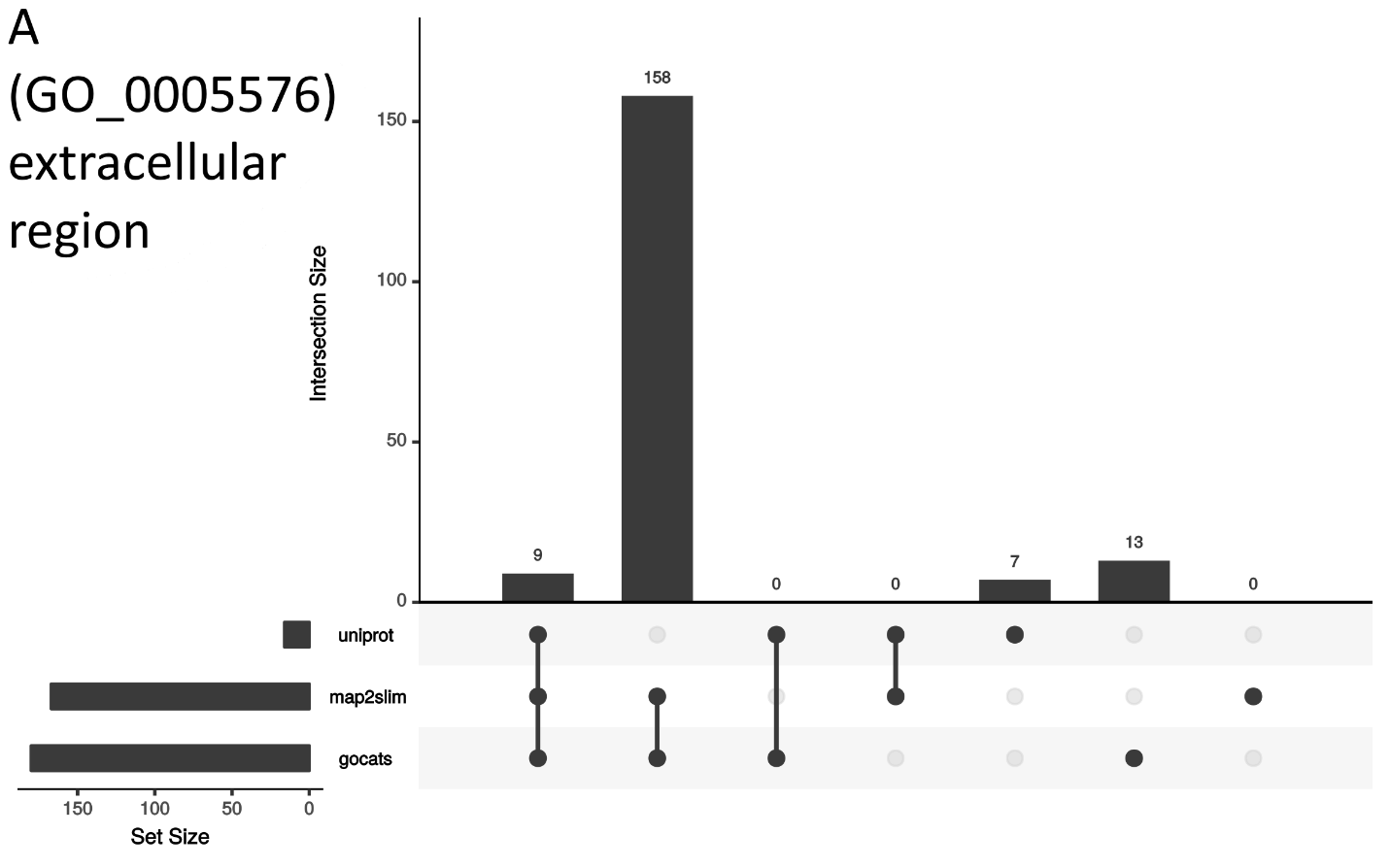
**


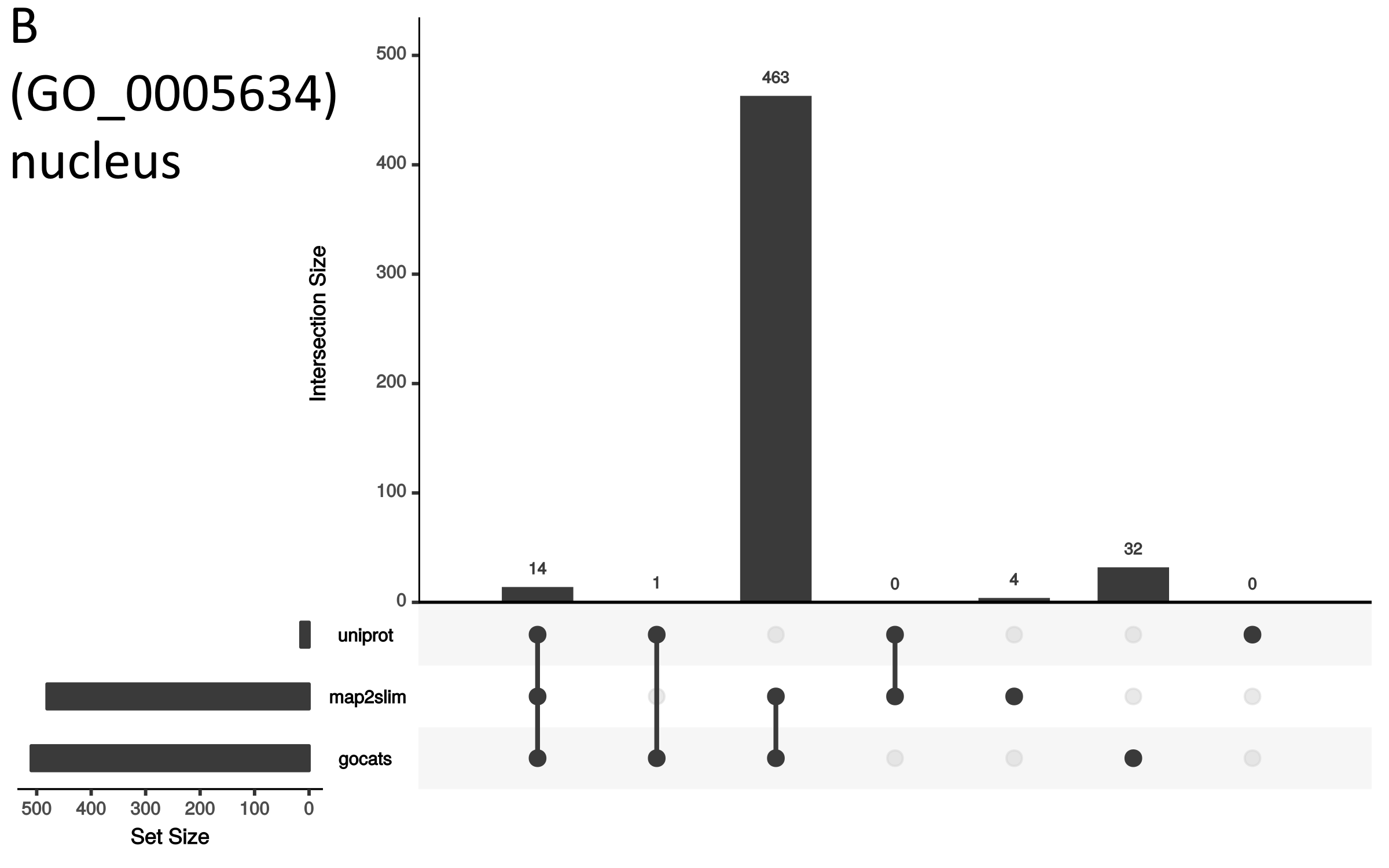

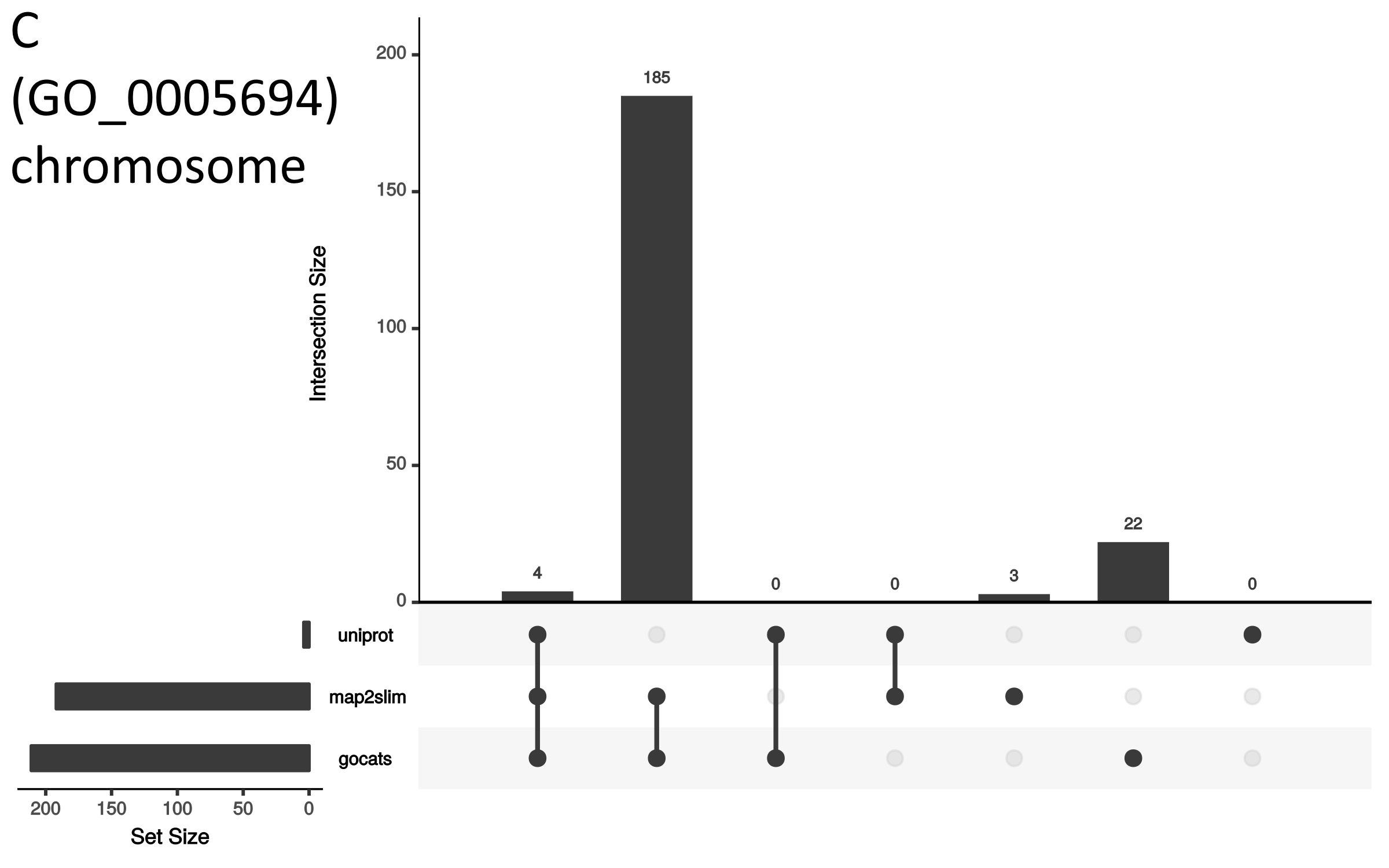

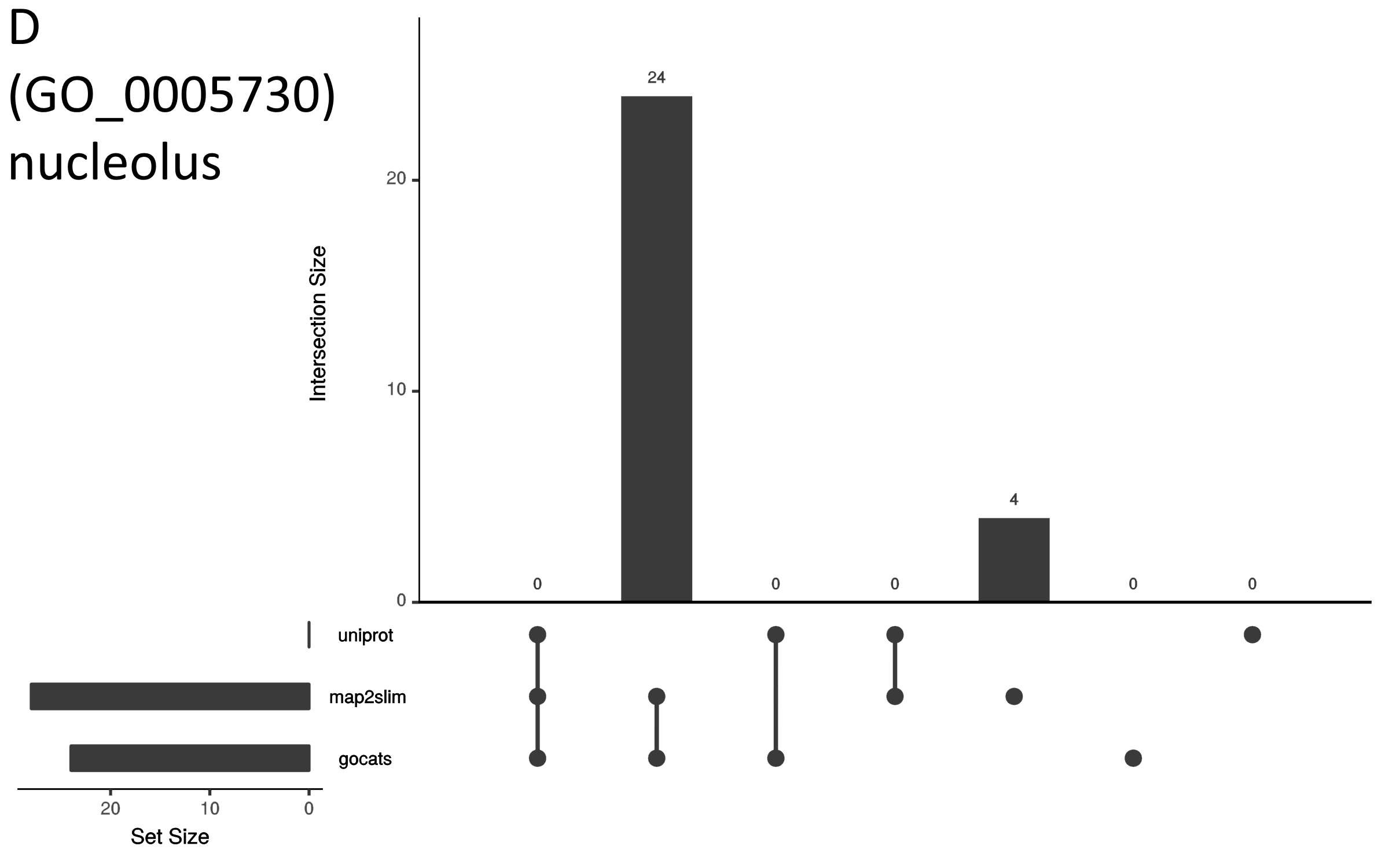

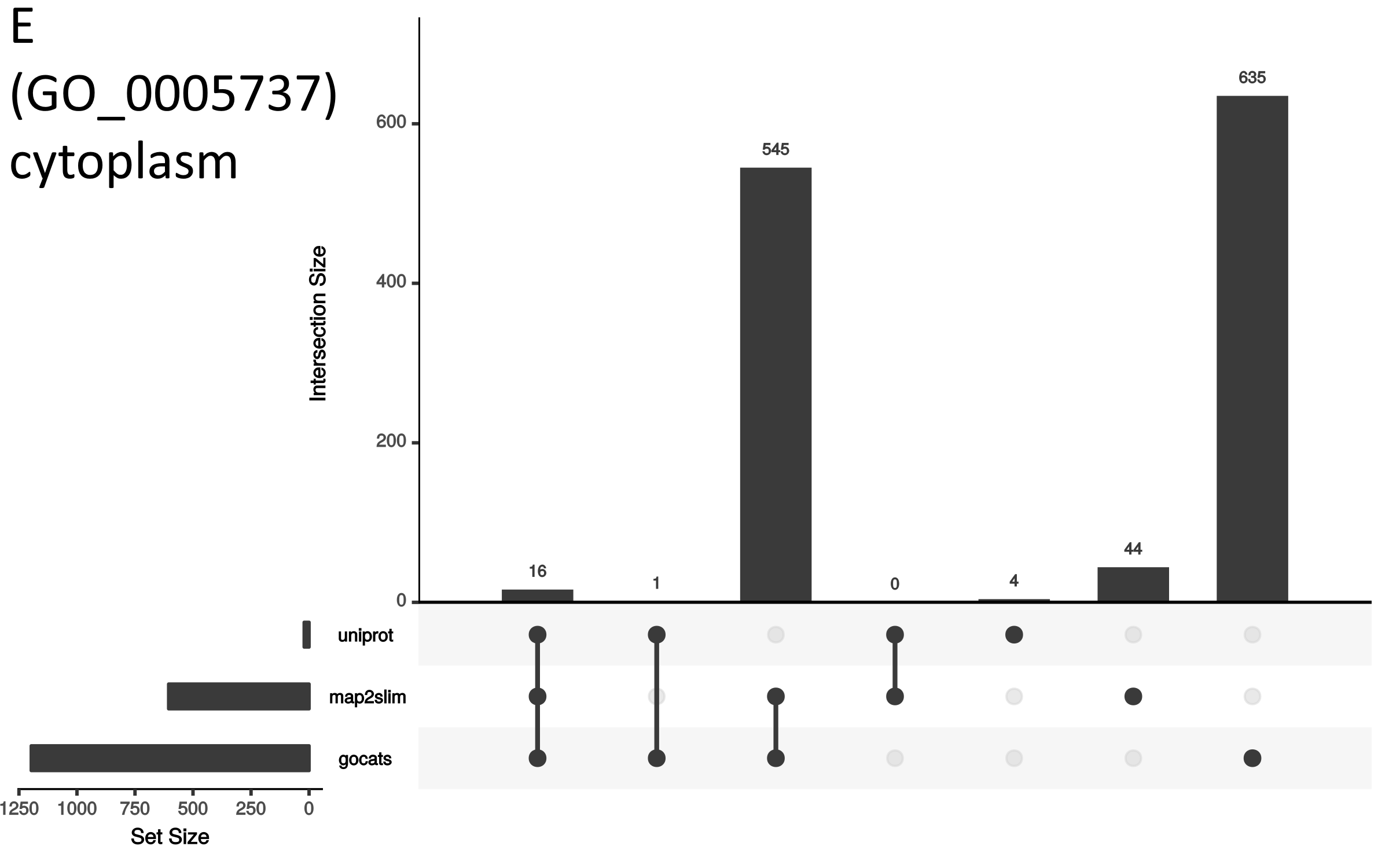

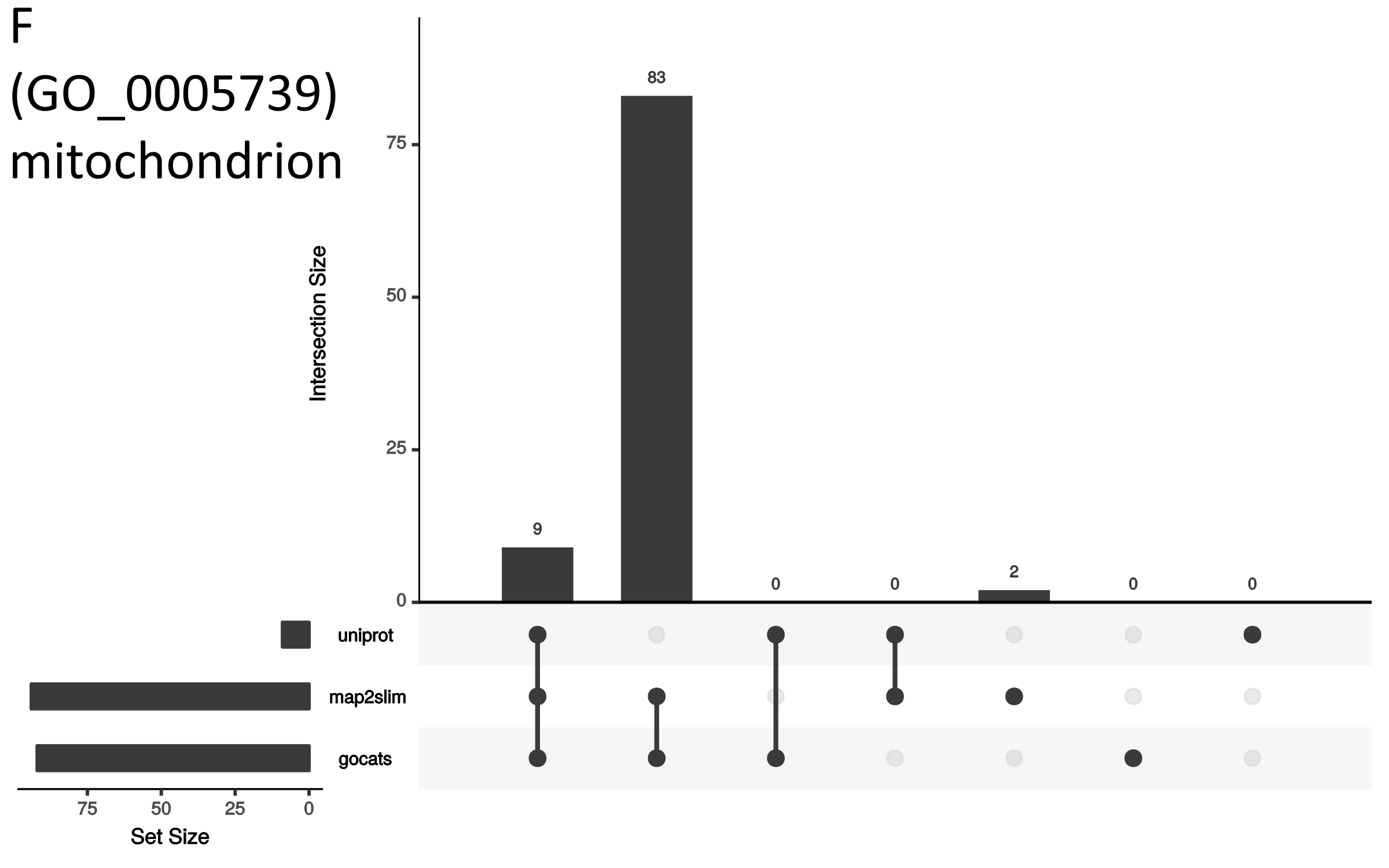

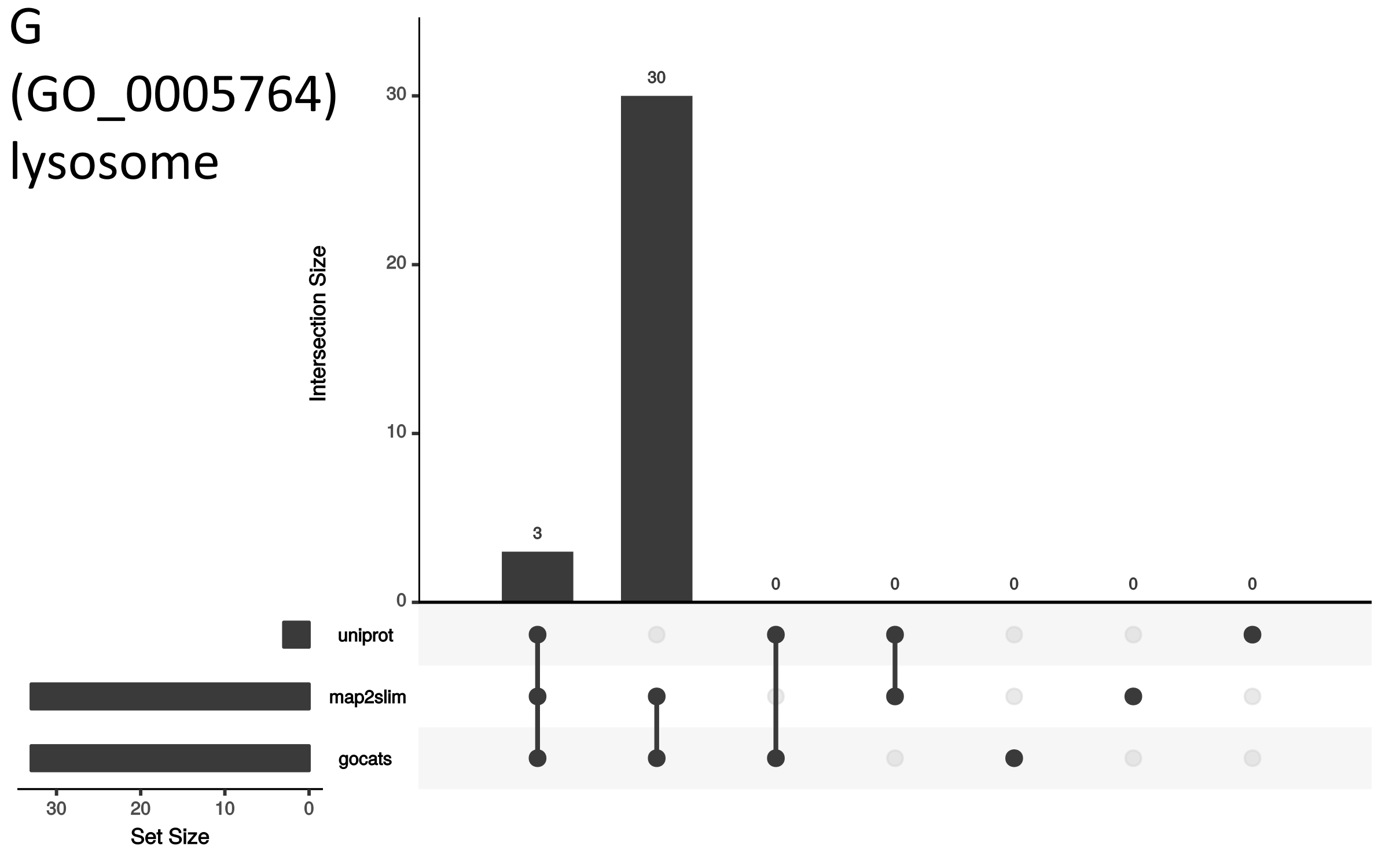

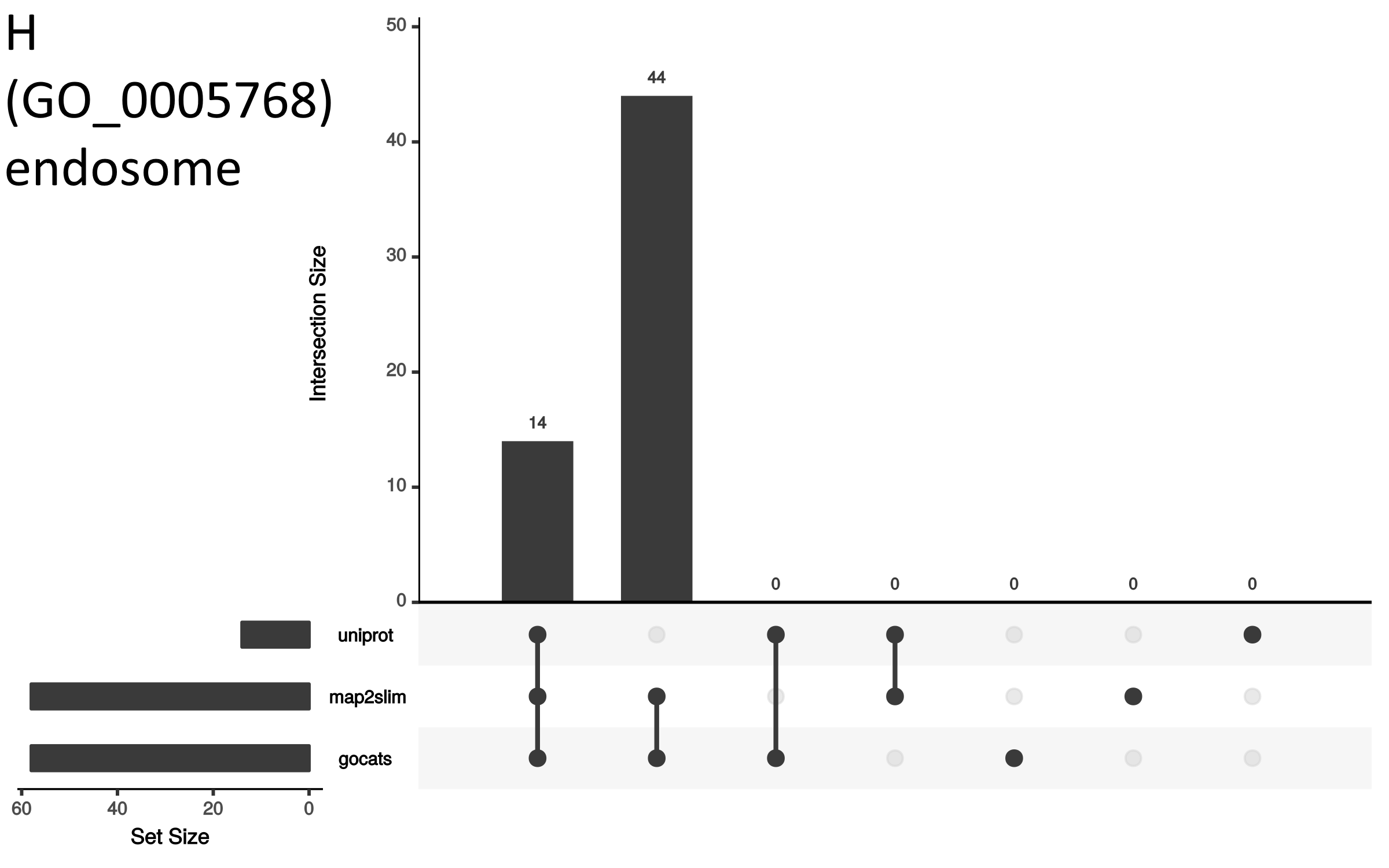

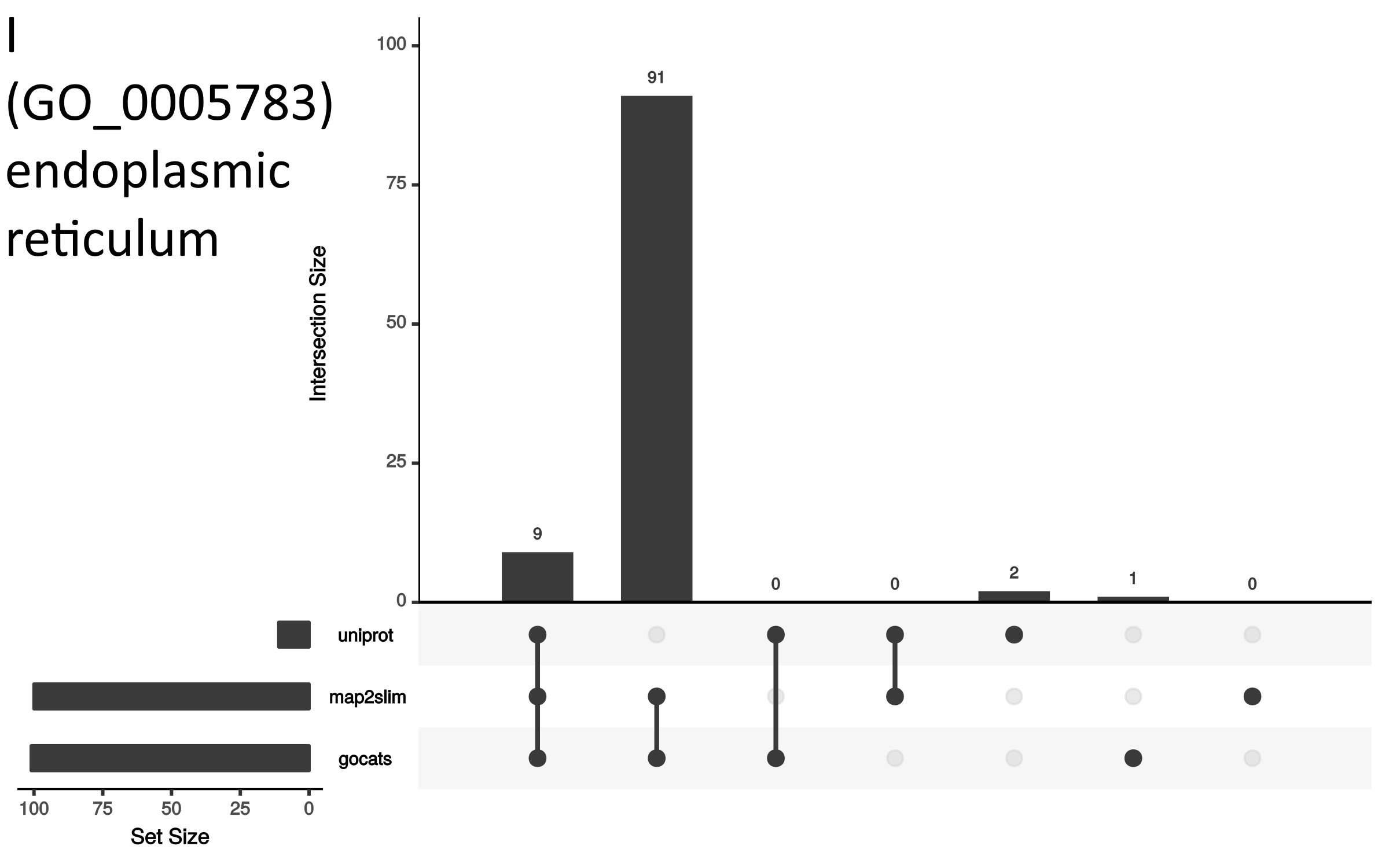

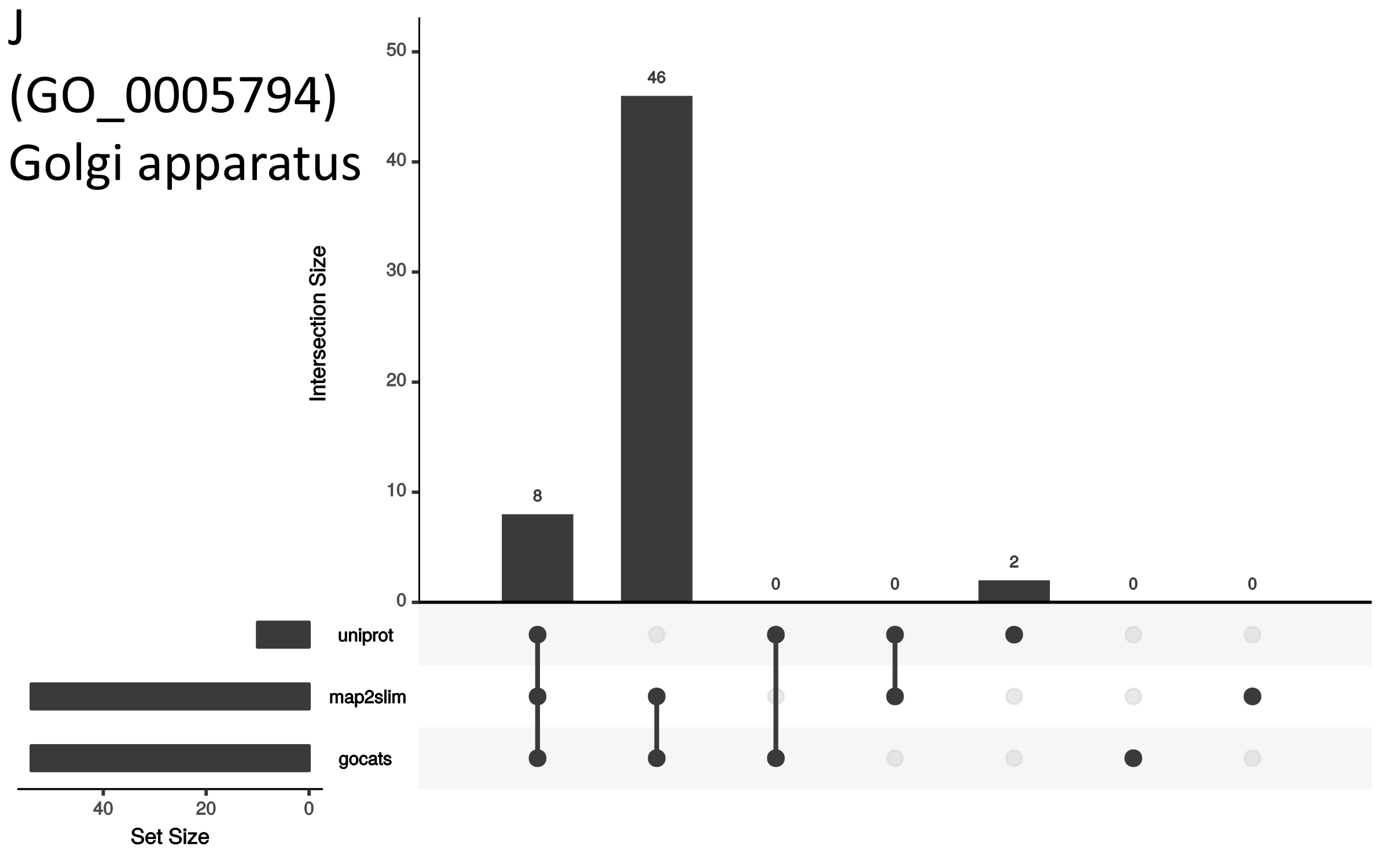

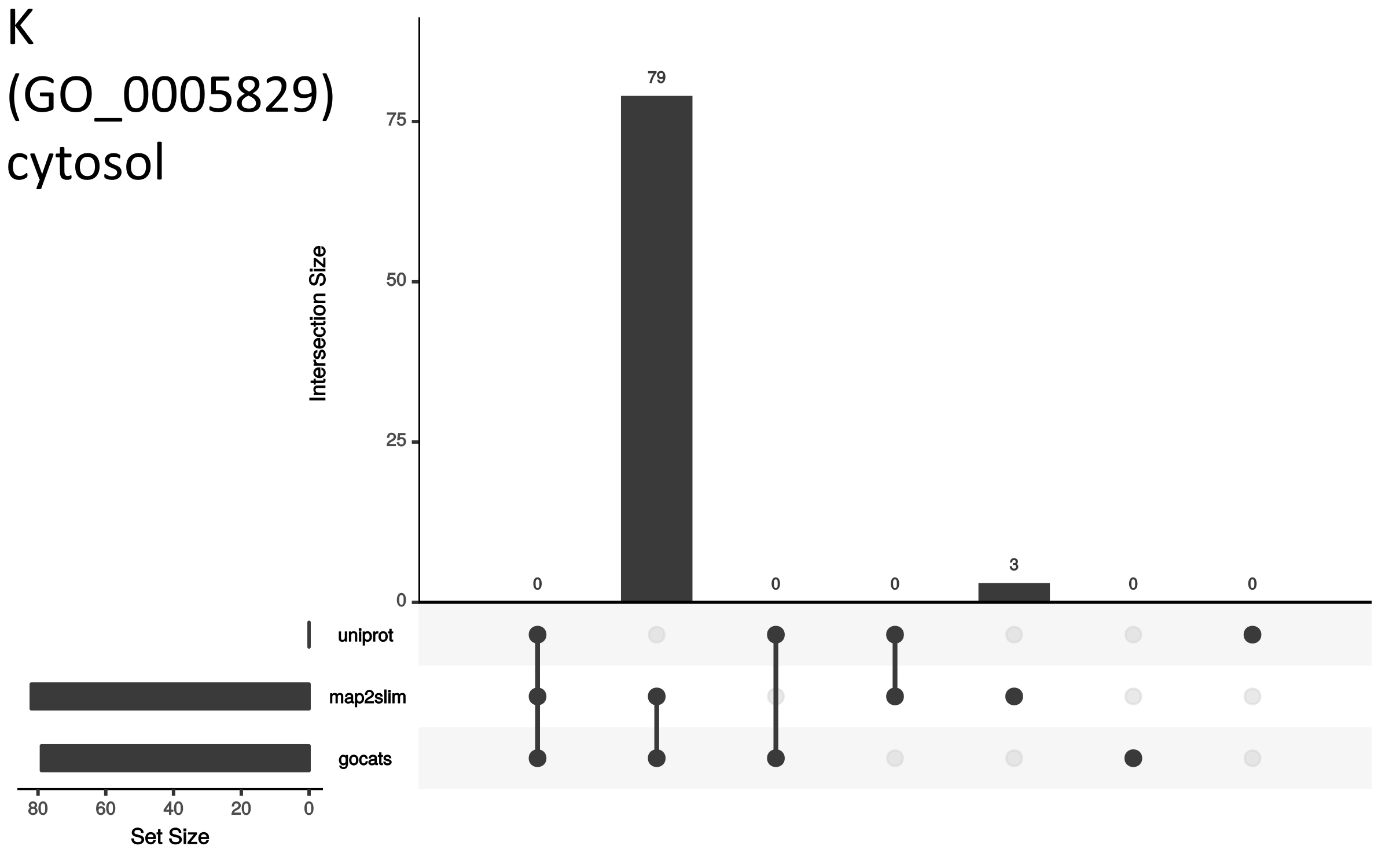

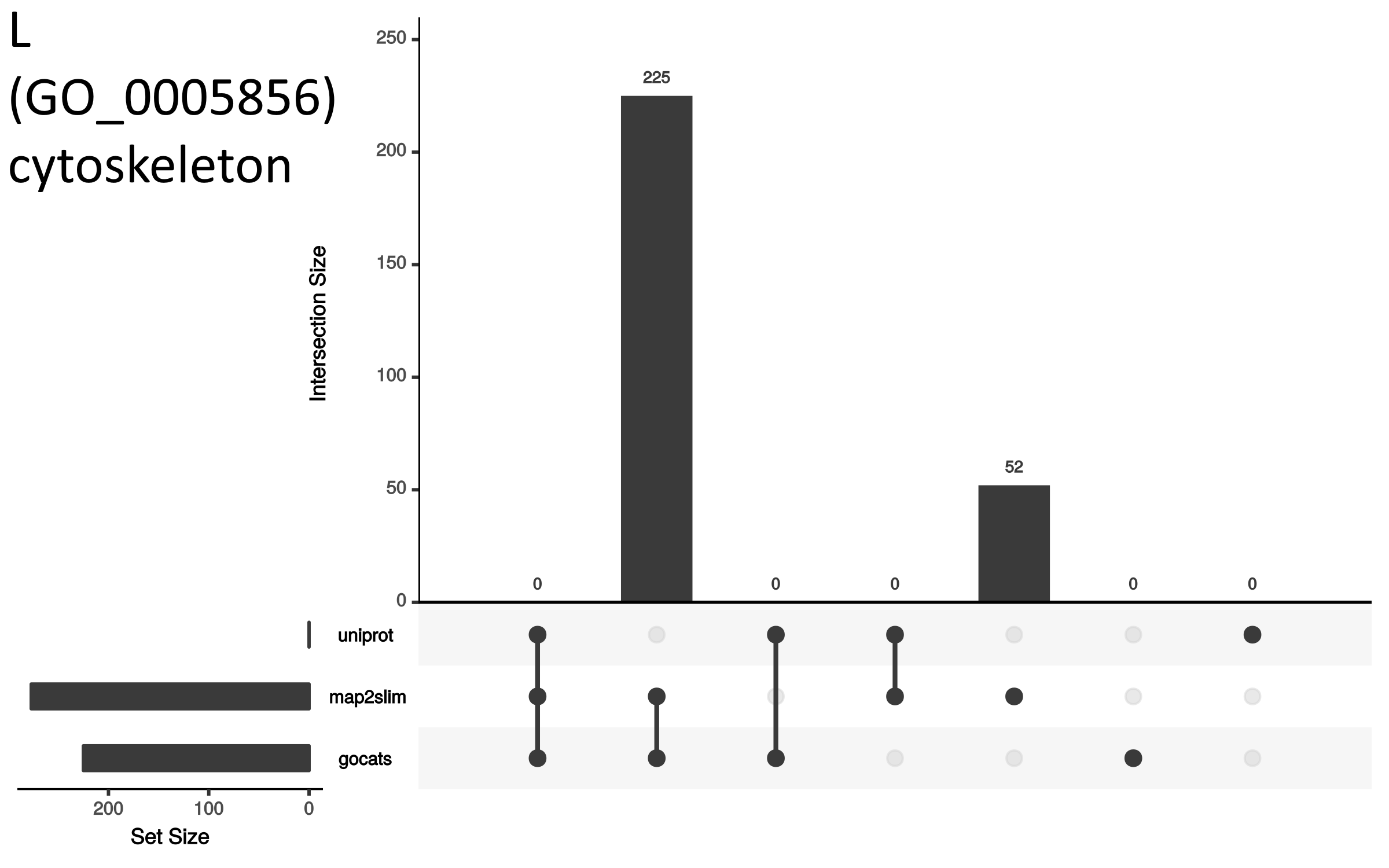

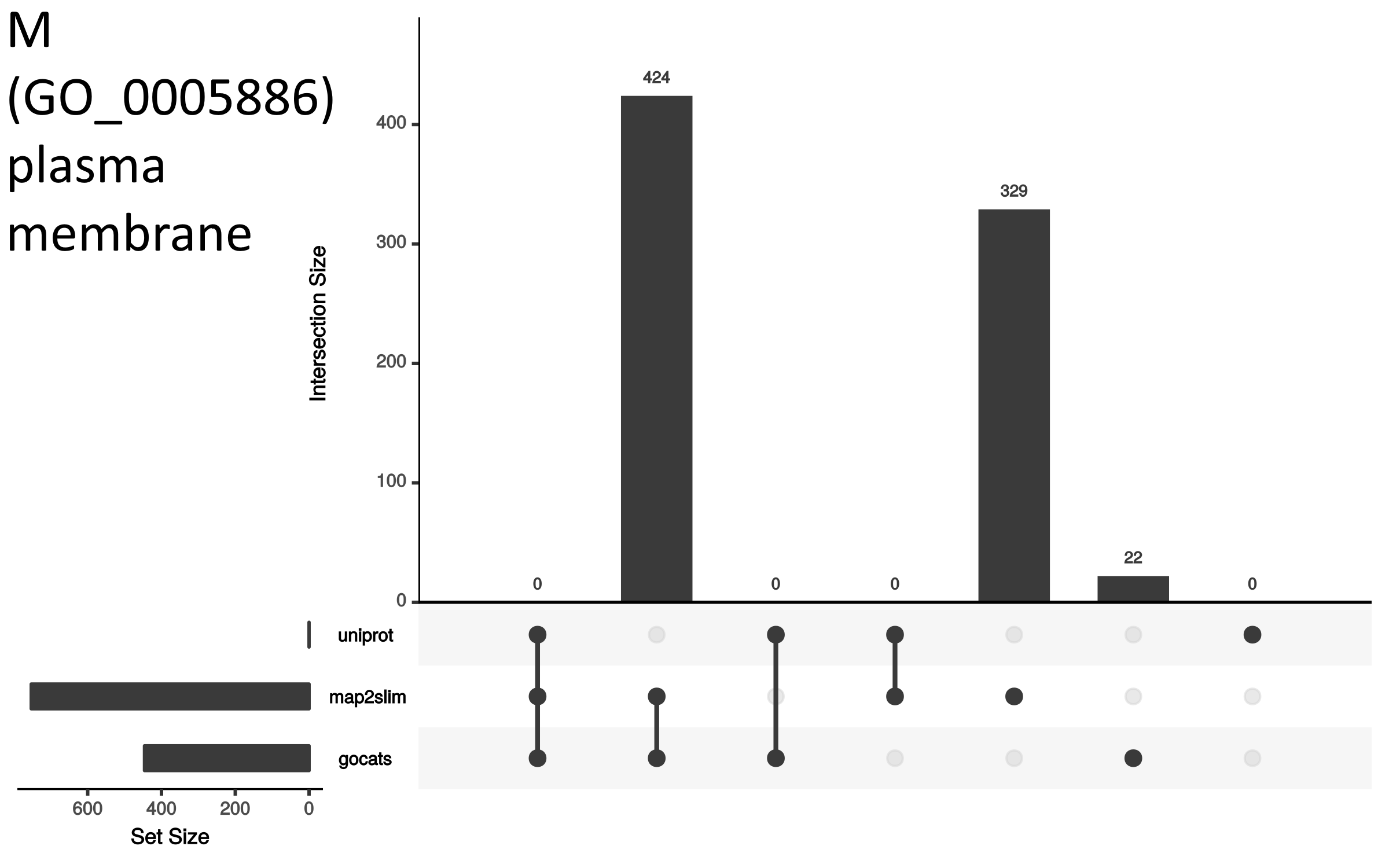

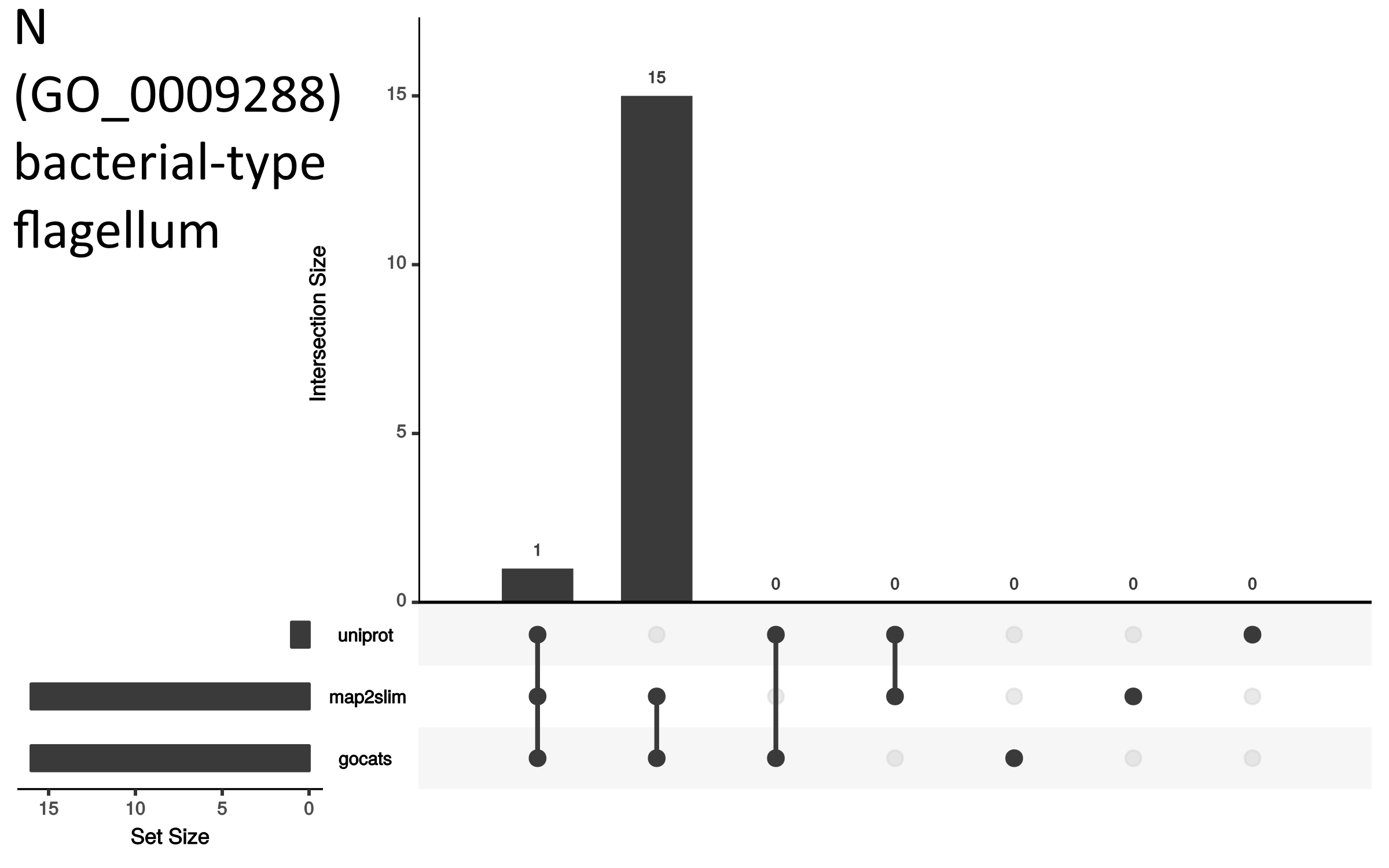

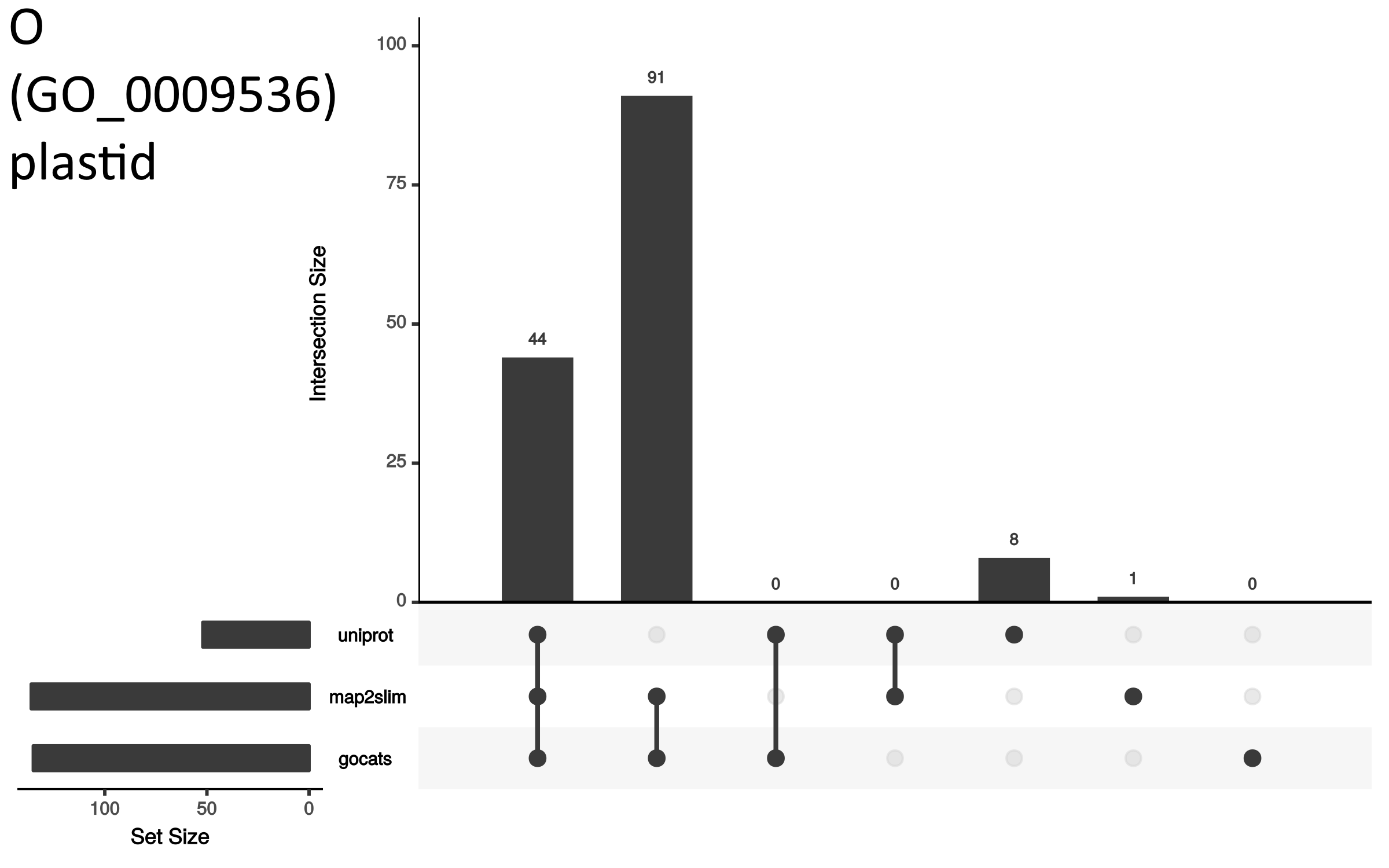

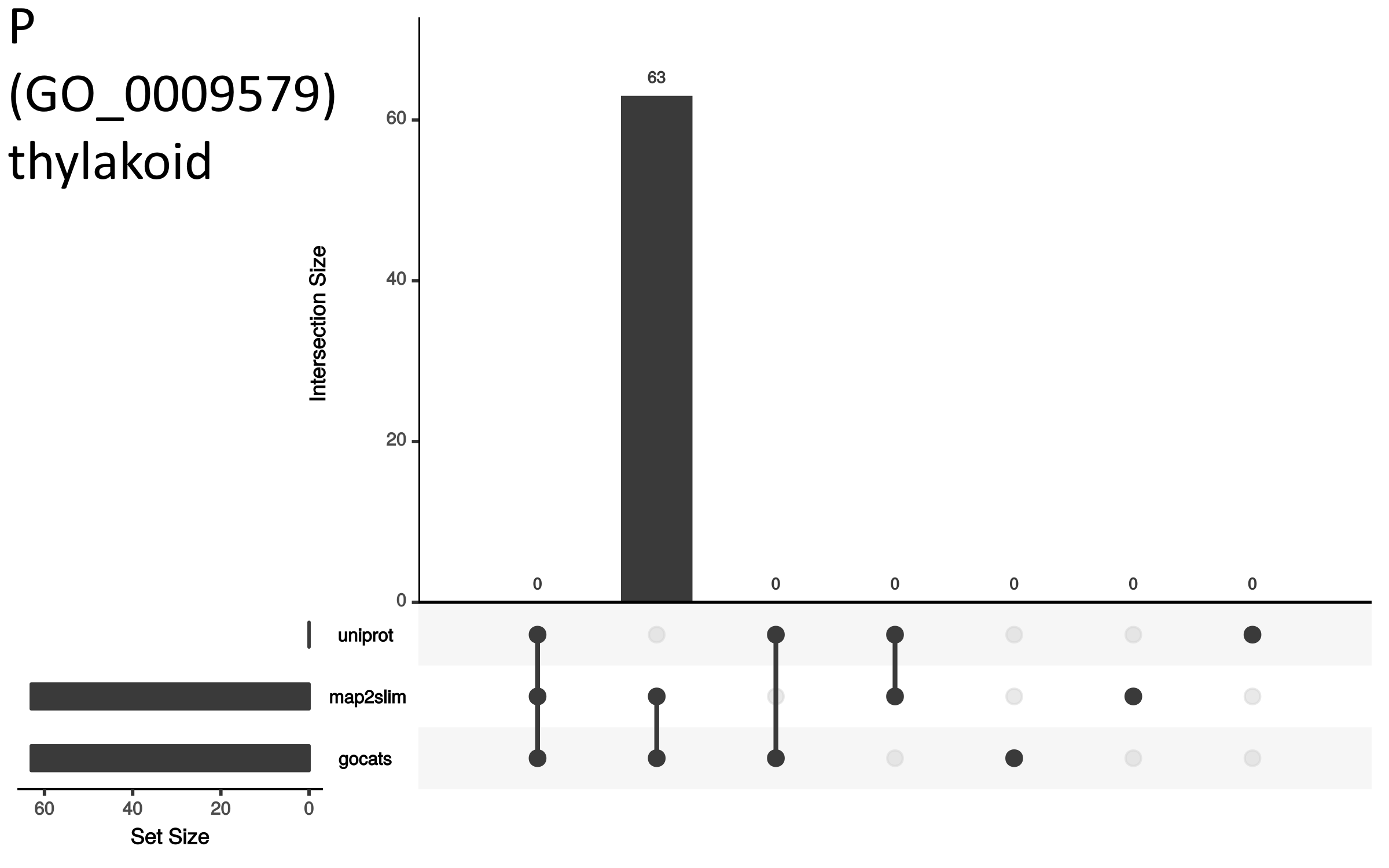

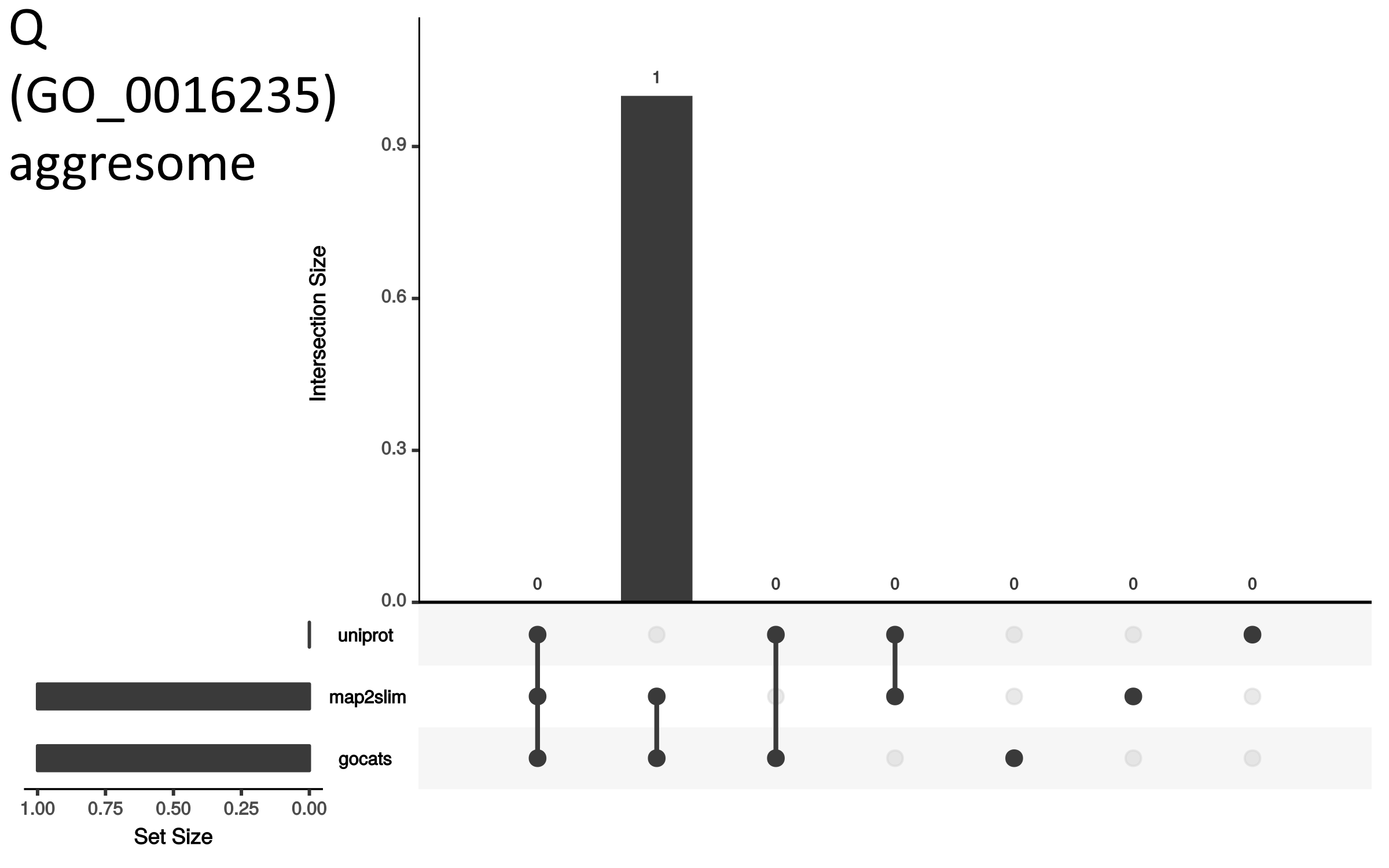

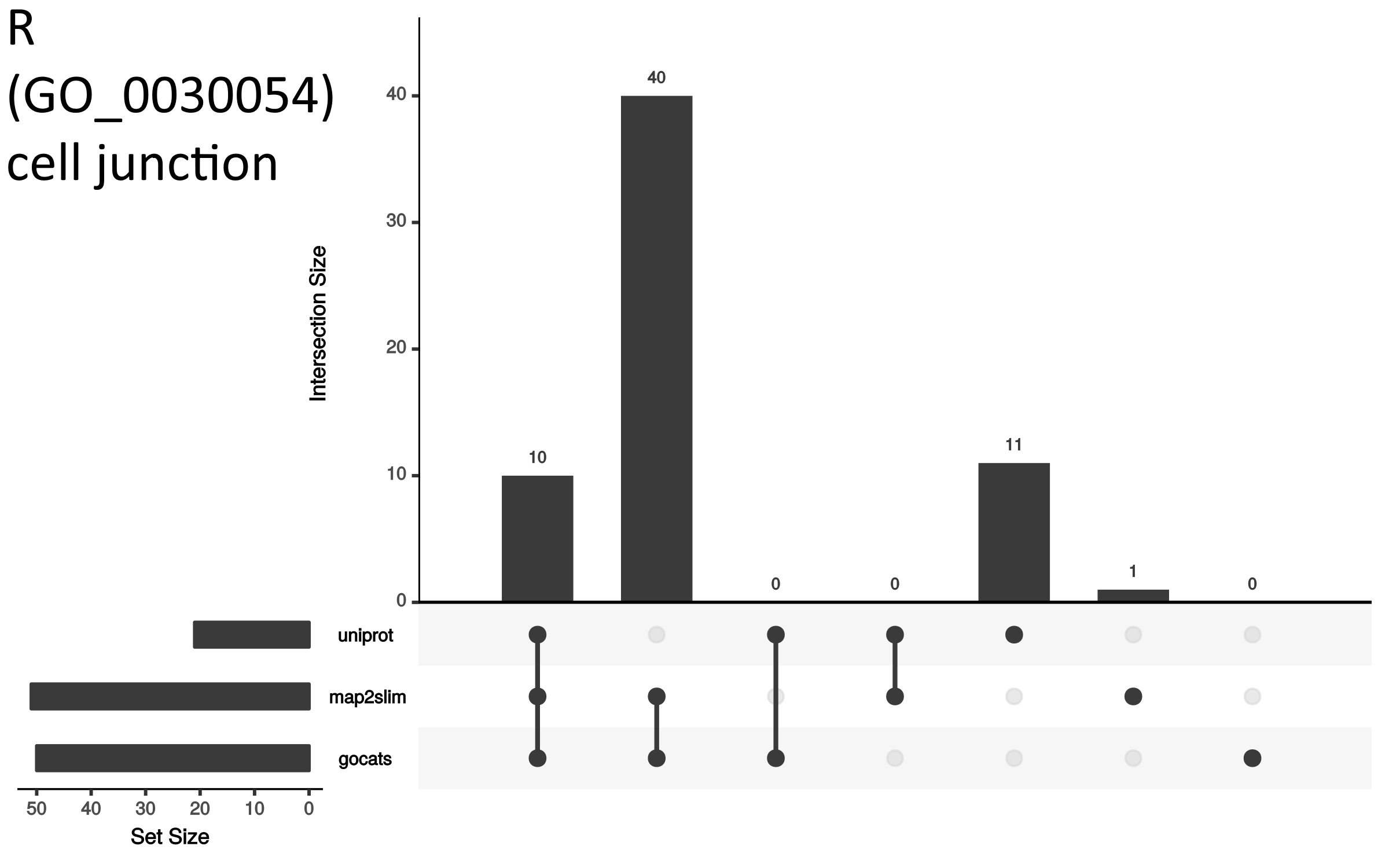

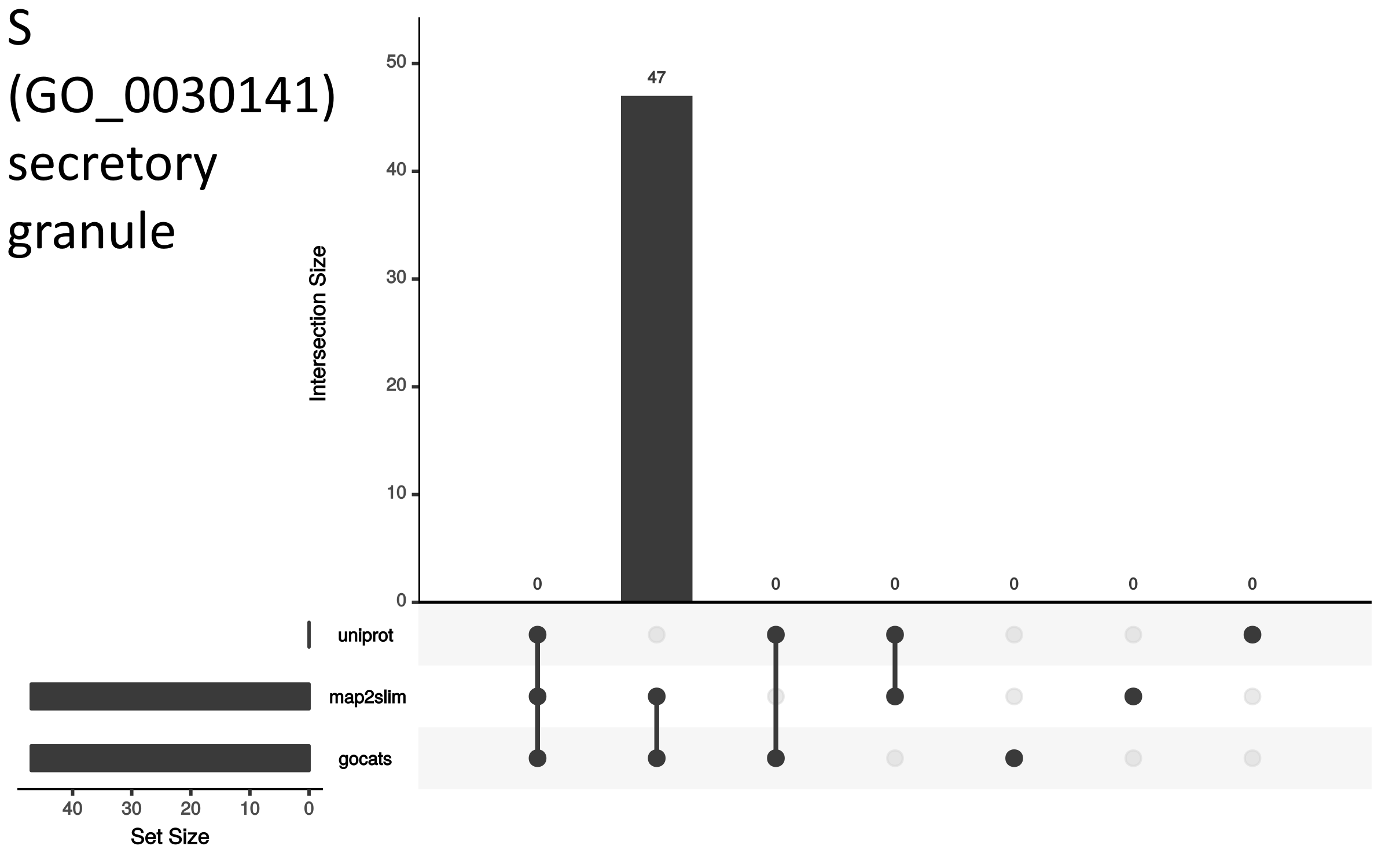

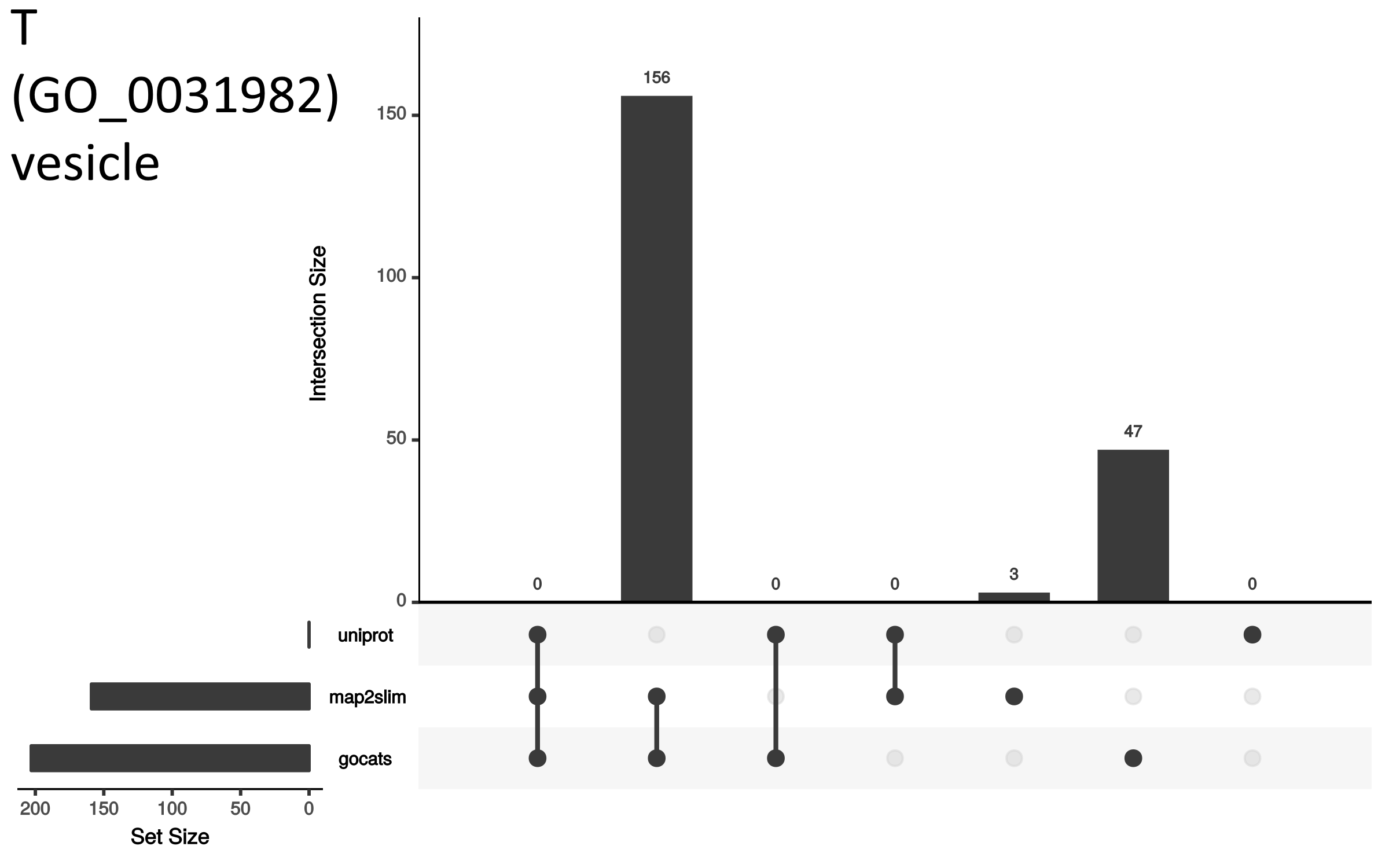

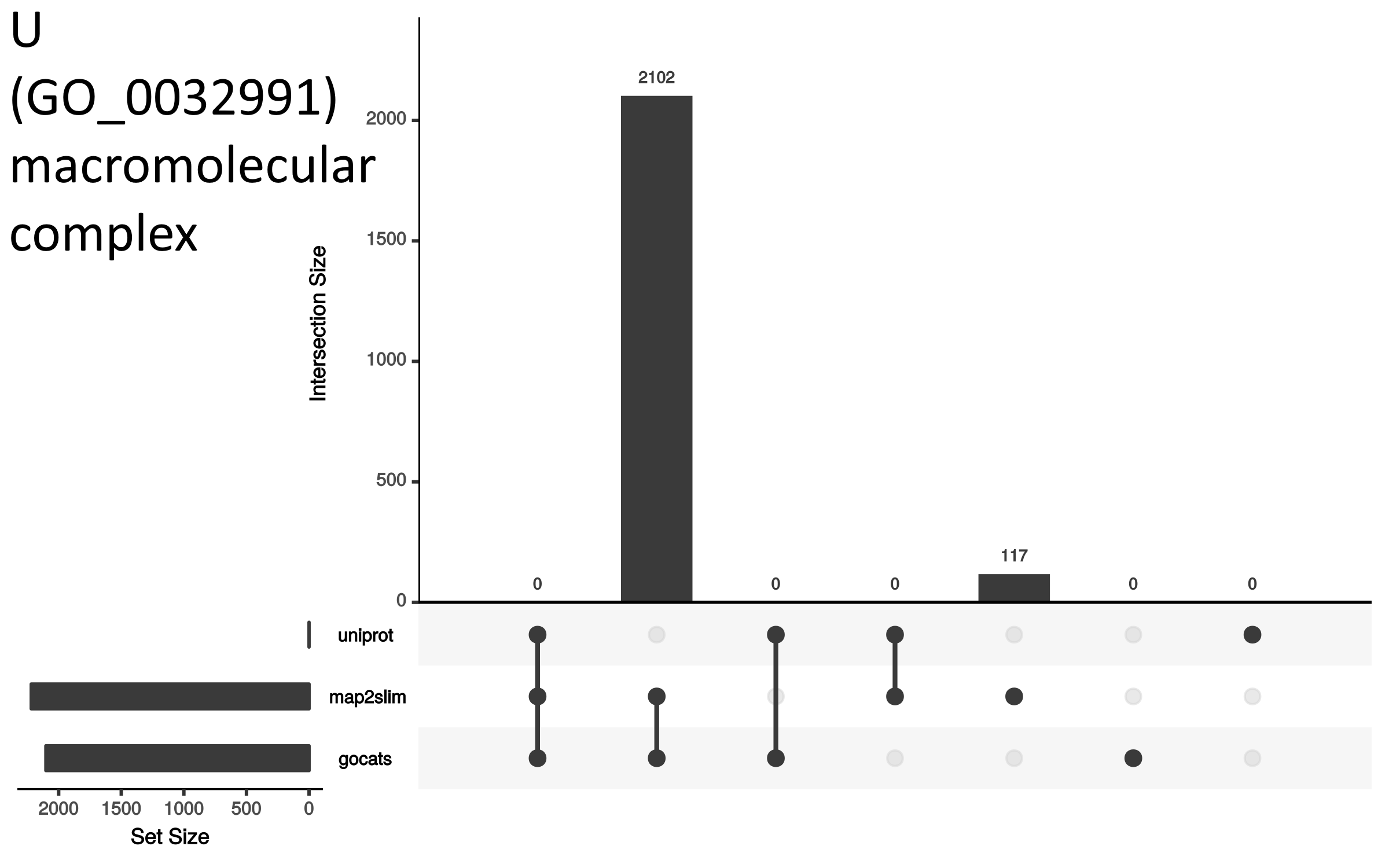

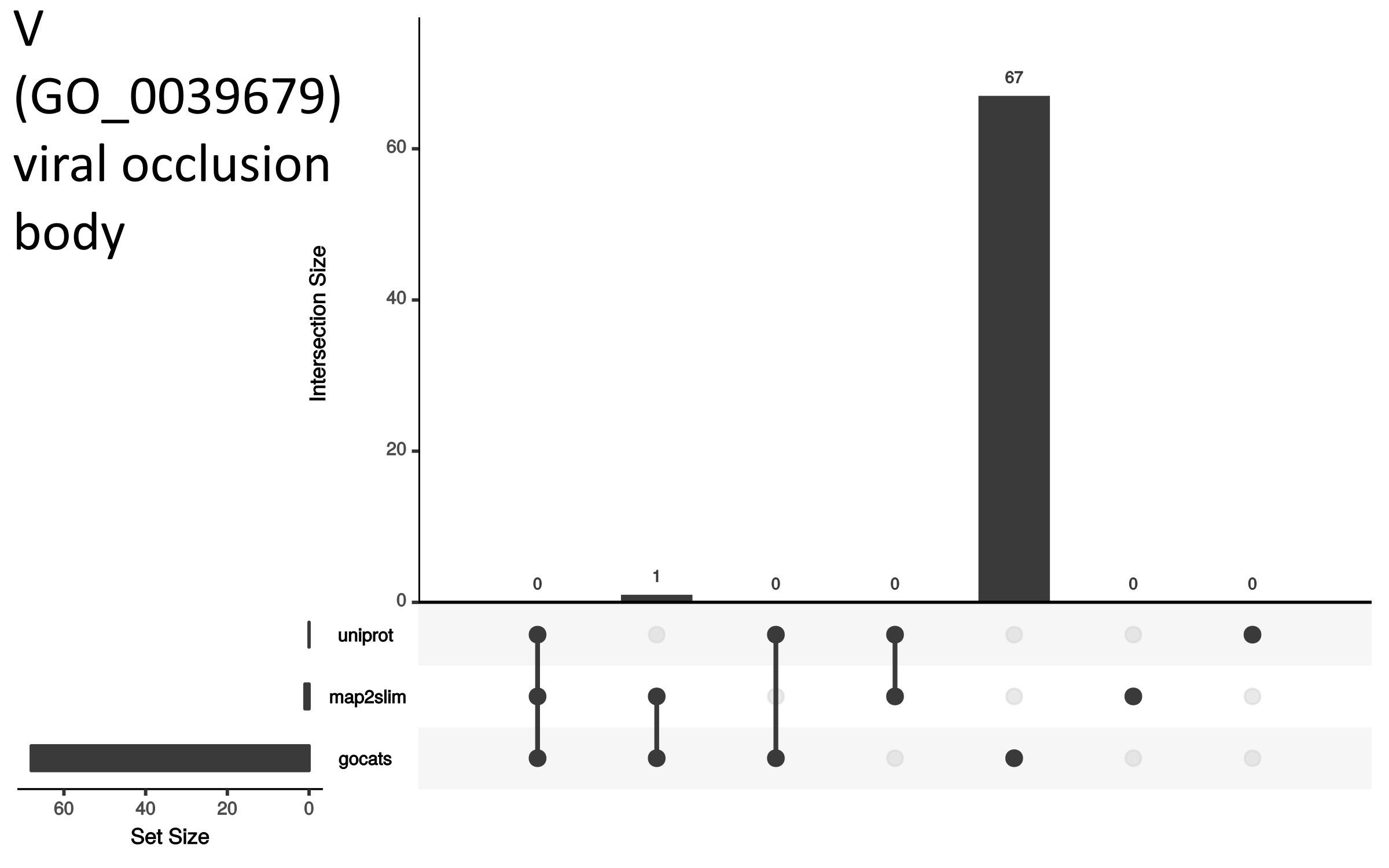

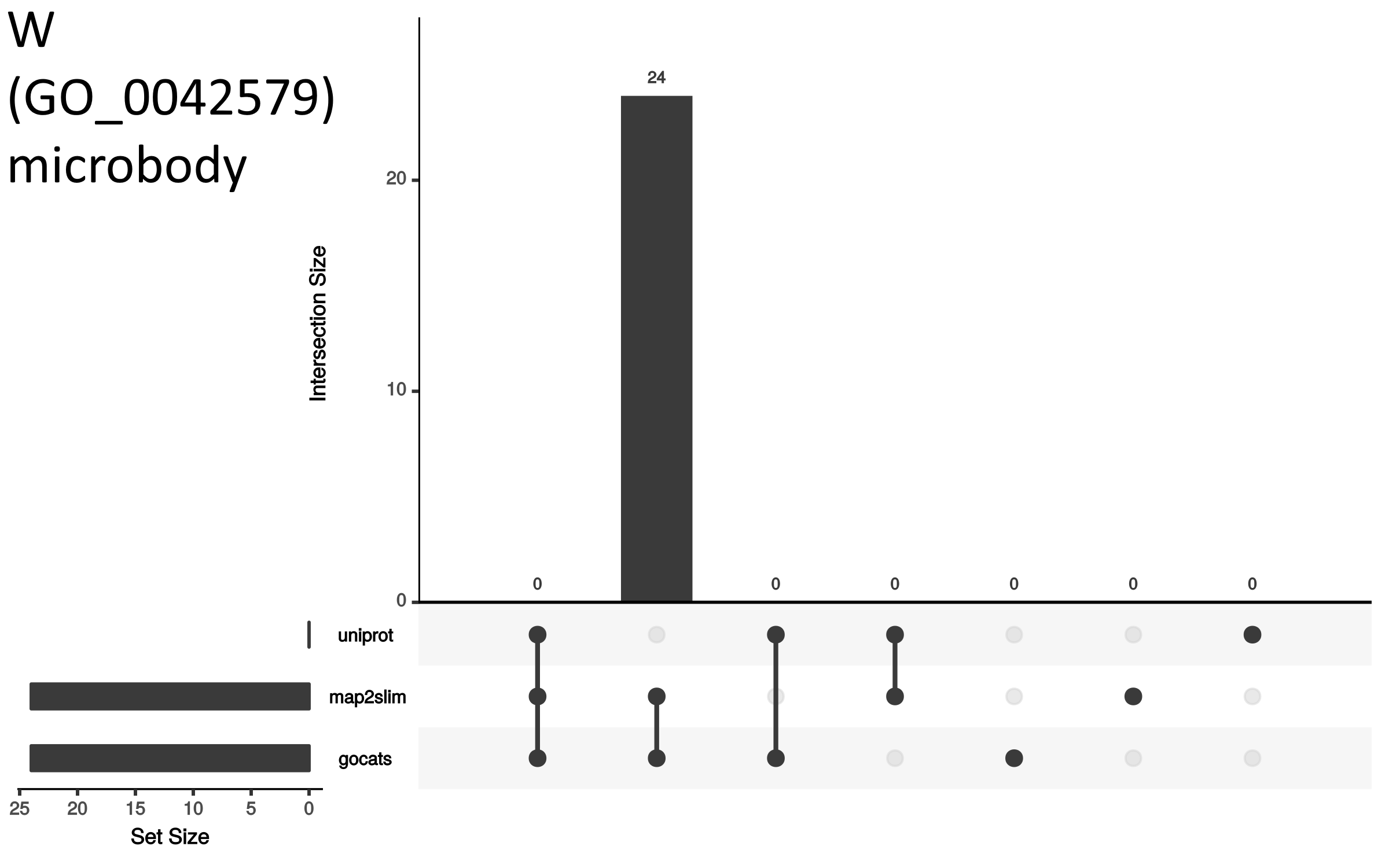

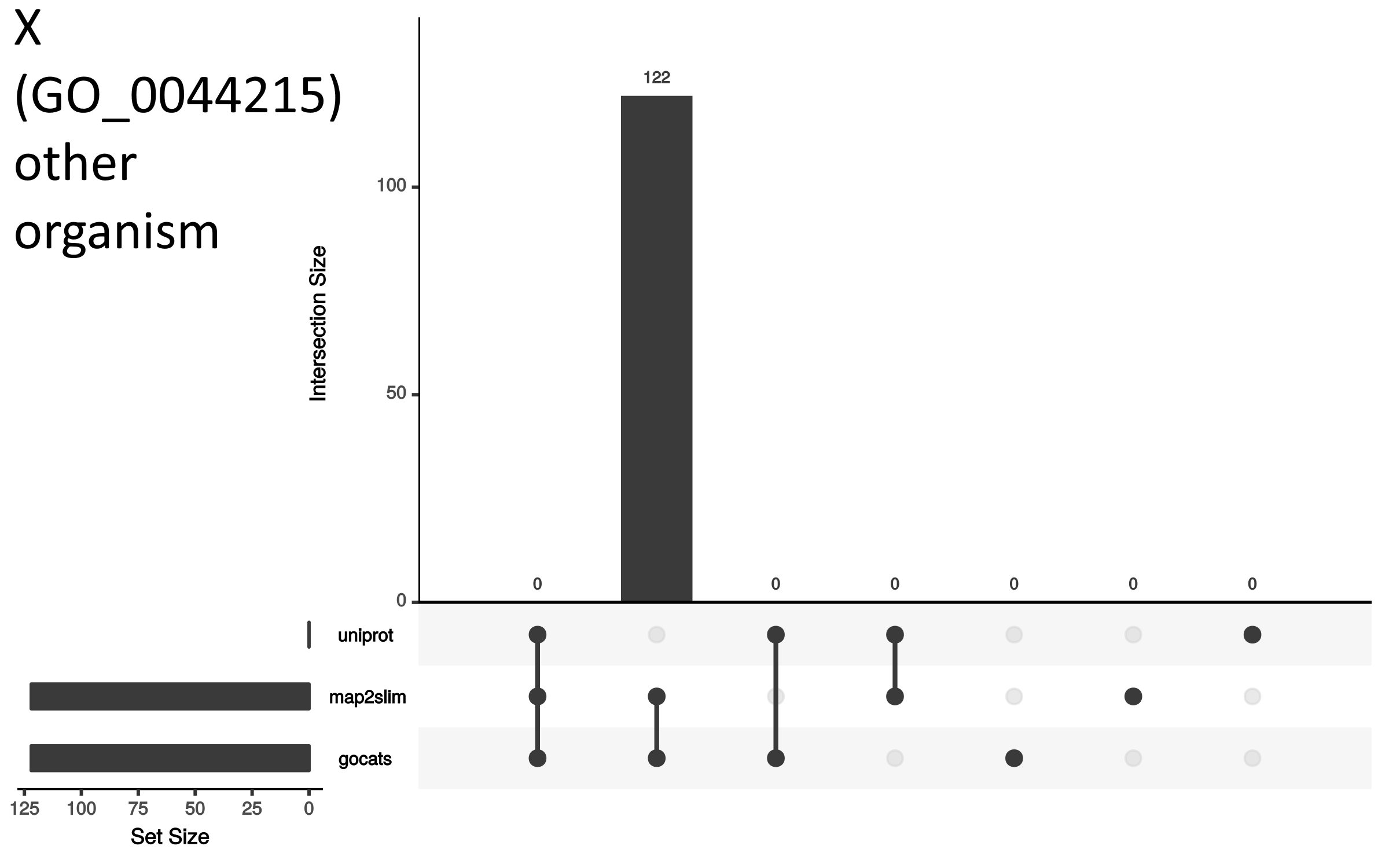

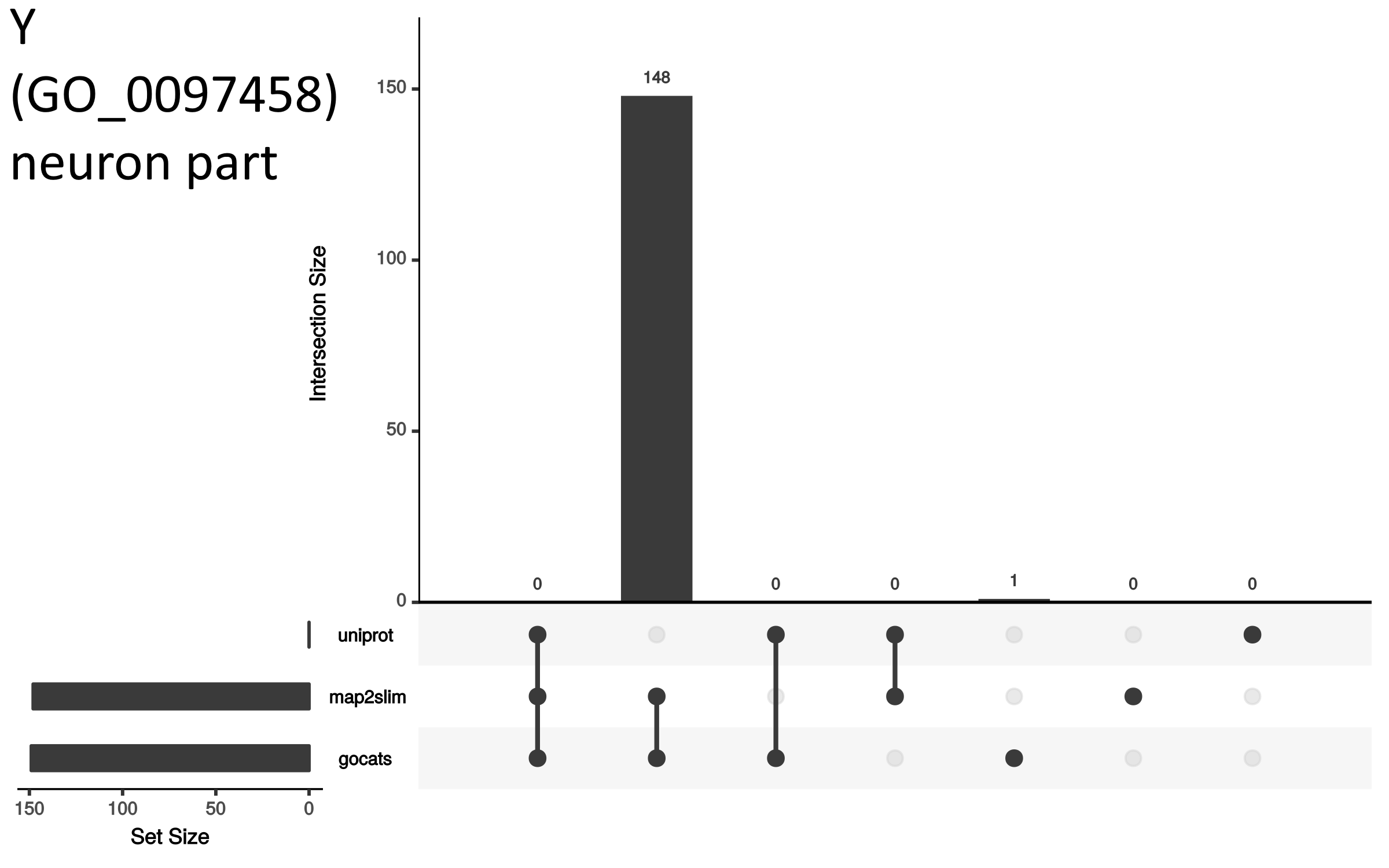

Supplement: S1 Data — (DOCX) [file pone.0233311.s001.docx]
