# Supplementary material for: GOcats: A tool for categorizing Gene Ontology into subgraphs of user-defined concepts
Source: PLoS One. 2020 Jun 11;15(6):e0233311. doi: 10.1371/journal.pone.0233311 (PMC7289357; doi:10.1371/journal.pone.0233311)
Supplement: S2 Data — (DOCX) [file pone.0233311.s002.docx]

**Supplementary Data 2 – List of GO terms mapped by Map2Slim to the term plasma membrane that were not mapped to this location by GOcats**

The following list was created by printing the difference between the list of terms mapped to GO:0005886 in the map2slim mapping file: _output_file/Map2SlimMapping.json_pickle and the list of terms mapped to GO:0005886 in the GOcats mapping file: _output_file/GC_content_mapping.json_pickle.

GO:0031240

GO:0070082

GO:0097544

GO:0044308

GO:0031308

GO:0036398

GO:0070381

GO:0032426

GO:0044614

GO:1990024

GO:0032421

GO:1990475

GO:0005642

GO:0031243

GO:0097386

GO:0032175

GO:0044326

GO:0070319

GO:0002180

GO:0072372

GO:0043005

GO:0097440

GO:0030175

GO:0098861

GO:0085035

GO:0043194

GO:0097658

GO:0097556

GO:0043197

GO:0097567

GO:0044096

GO:0009419

GO:0033270

GO:0060203

GO:0036157

GO:0001520

GO:0042175

GO:0031229

GO:0035686

GO:0097609

GO:0097545

GO:0097226

GO:0031245

GO:0097559

GO:1990805

GO:0030424

GO:0098860

GO:0097541

GO:1902673

GO:1990476

GO:0097444

GO:0044327

GO:0031246

GO:1990462

GO:1990717

GO:0031965

GO:0032433

GO:0002140

GO:0090396

GO:0090395

GO:0061177

GO:0097543

GO:0005937

GO:0034993

GO:0044316

GO:0044302

GO:0031521

GO:0098538

GO:0044393

GO:0097454

GO:1902672

GO:0090406

GO:0097539

GO:0036038

GO:1990906

GO:0031514

GO:0097229

GO:1990876

GO:1990474

GO:0097451

GO:1990795

GO:0044301

GO:0031242

GO:0097563

GO:0001726

GO:0012507

GO:0005643

GO:0097565

GO:0098568

GO:0012505

GO:1990021

GO:1990719

GO:0035996

GO:0097511

GO:0097557

GO:0009279

GO:0031241

GO:0016028

GO:0005640

GO:0036405

GO:0036418

GO:0044463

GO:0097455

GO:0031316

GO:0030905

GO:1990635

GO:0031080

GO:1990716

GO:0044295

GO:0001534

GO:0001536

GO:1901589

GO:0097463

GO:0097629

GO:0097649

GO:0071212

GO:0031563

GO:0032839

GO:0097542

GO:0005858

GO:0061176

GO:0090397

GO:0060091

GO:0060200

GO:0002142

GO:0035230

GO:0043203

GO:0030127

GO:0005639

GO:1990862

GO:0060199

GO:0097540

GO:0070081

GO:0097442

GO:0034992

GO:0097014

GO:1901588

GO:0097449

GO:1903349

GO:1902677

GO:0044294

GO:0030288

GO:0031230

GO:0071598

GO:0071683

GO:1902675

GO:1902636

GO:1990025

GO:0005902

GO:0097608

GO:0097558

GO:0070685

GO:0005931

GO:0044300

GO:0085041

GO:0090552

GO:1990761

GO:0032838

GO:0036421

GO:1902671

GO:0061175

GO:0097554

GO:0070451

GO:0070762

GO:1990435

GO:1990796

GO:0044292

GO:0097560

GO:0070852

GO:0044613

GO:0001750

GO:0030906

GO:0098859

GO:0097537

GO:0097516

GO:1902676

GO:0097447

GO:1902737

GO:0005929

GO:0031912

GO:0044317

GO:0097462

GO:0020016

GO:0005879

GO:0044611

GO:0097465

GO:1990718

GO:0009289

GO:0045203

GO:1990905

GO:0097561

GO:0044441

GO:0097630

GO:0044309

GO:1990769

GO:0097448

GO:0035869

GO:0030134

GO:0031244

GO:0097566

GO:0044303

GO:0005637

GO:0060201

GO:0044304

GO:0060202

GO:0044224

GO:1990030

GO:0071808

GO:1902674

GO:0030993

GO:0001400

GO:0097225

GO:0033268

GO:0031513

GO:0097464

GO:0030313

GO:0061174

GO:0042995

GO:1990031

GO:0097441

GO:0044612

GO:0036194

GO:0035618

GO:0035619

GO:0044306

GO:0032162

GO:0044443

GO:0043195

GO:0097456

GO:0030133

GO:1990033

GO:0098862

GO:0001931

GO:0097653

GO:0005903

GO:0097228

GO:0036420

GO:1990434

GO:0031143

GO:0097227

GO:0033267

GO:0030425

GO:0044615

GO:0044307

GO:0097564

GO:0031512

GO:0030904

GO:0032420

GO:0036064

GO:0098836

GO:0042757

GO:1904115

GO:0043198

GO:0044570

GO:0005635

GO:0097450

GO:1990900

GO:0031309

GO:0044447

GO:0002141

GO:0035253

GO:0090553

GO:0097467

GO:1990819

GO:0005641

GO:0036126

GO:1990623

GO:0097457

GO:1990026

GO:0097589

GO:0044453

GO:0044293

GO:0098539

GO:0044195

GO:0044305

GO:0030426

GO:0097631

GO:0009418

GO:0097555

GO:0001535

GO:0090404

GO:0032391

GO:0030658

GO:0043196

GO:0032059

GO:0098788

GO:0036407

GO:0070687

GO:1902678

GO:1990027

GO:0070083

GO:0071437

GO:0032123

GO:0043332

GO:0033269

GO:0044299

GO:0098566

GO:0098858

GO:1990032

GO:0098846

GO:0090405

GO:0070382

GO:0098854

GO:0044296

GO:0030027

GO:0097481

GO:0002139

GO:0097546

GO:1990720

GO:0009276

GO:0005930

GO:0036406

GO:1990427

GO:1990812

GO:0036419

GO:1990063

GO:0097650

GO:0097224

GO:0060204

GO:1990875

GO:0071595

GO:1990909

GO:1990612

GO:0036156

GO:0005905

GO:1990020

GO:0043679

GO:0097387

GO:0097562
