# Supplementary material for: GOcats: A tool for categorizing Gene Ontology into subgraphs of user-defined concepts
Source: PLoS One. 2020 Jun 11;15(6):e0233311. doi: 10.1371/journal.pone.0233311 (PMC7289357; doi:10.1371/journal.pone.0233311)
Supplement: S1 File — (DOCX) [file pone.0233311.s003.docx]

All figures and supplementary data are available at <https://figshare.com/s/21defede0cd3865742d4>.
